# Vacuum-Assisted Wound Closure with Mesh-Mediated Fascial Traction Achieves Better Outcomes than Vacuum-Assisted Wound Closure Alone: A Comparative Study

**DOI:** 10.1007/s00268-017-4354-3

**Published:** 2017-11-16

**Authors:** Giuseppe Salamone, Leo Licari, Giovanni Guercio, Albert Comelli, Mirko Mangiapane, Nicolò Falco, Roberta Tutino, Noemi Bagarella, Sofia Campanella, Calogero Porrello, Roberto Gullo, Gianfranco Cocorullo, Gaspare Gulotta

**Affiliations:** 1 General and Emergency Surgery – Policlinico P. Giaccone, University of Palermo, Via Liborio Giuffré 5, 90127 Palermo, Italy; 2 Department of Industrial and Digital Innovation, Policlinico P. Giaccone, University of Palermo, Via Liborio Giuffré 5, 90127 Palermo, Italy

## Abstract

**Background:**

Open abdomen (OA) permits the application of damage control surgery principles when abdominal trauma, sepsis, severe acute peritonitis and abdominal compartmental syndrome (ACS) occur.

**Methods:**

Non-traumatic patients treated with OA between January 2010 and December 2015 were identified in a prospective database, and the data collected were retrospectively reviewed. Patients’ records were collected from charts and the surgical and intensive care unit (ICU) registries. The Acosta “*modified*” technique was used to achieve fascial closure in *vacuum*-*assisted wound closure and mesh*-*mediated fascial traction* (VAWCM) patients. Sex, age, simplified acute physiology score II (SAPS II), abdominal compartmental syndrome (ACS), cardiovascular disease (CVD) and surgical technique performed were evaluated in a multivariate analysis for mortality and fascial closure prediction.

**Results:**

Ninety-six patients with a median age of 69 (40–78) years were included in the study. Sixty-nine patients (72%) underwent VAWCM. Forty-one patients (68%) achieved primary fascia closure: two patients (5%) were treated with VAWC (37 median days) versus 39 patients (95%) who were treated with VAWCM (10 median days) (*p* = 0.0003). Forty-eight patients underwent OA treatment due to ACS, and 24 patients (50%) survived compared to 36 patients (75%) from the “other reasons” group (*p* = 0.01). The ACS group required longer mechanical ventilator support (*p* = 0.006), length of stay in hospital (*p* = 0.005) and in ICU (*p* = 0.04) and had higher SAPS II scores (*p* = 0.0002).

**Conclusions:**

The survival rate was 62%. ACS (*p* = 0.01), SAPS II (*p* = 0.004), sex (*p* = 0.01), pre-existing CVD (*p* = 0.0007) and surgical technique (VAWC vs VAWCM) (*p* = 0.0009) were determined to be predictors of mortality. Primary fascial closure was obtained in 68% of cases. VAWCM was found to grant higher survival and primary fascial closure rate.

## Introduction

Temporary abdominal closure (TAC) is the easiest way to facilitate re-operations when needed, such as in secondary and tertiary peritonitis or acute haemorrhagic necrotic pancreatitis or trauma.

Contemporary use of negative pressure therapy (NPT) allows the reduction in bacterial load and pro-inflammatory cytokines [1–6]. It also allows the standardisation of open abdomen (OA) techniques, improving prognosis and results of the procedure [6–9].

Recently, useful innovations in OA techniques have been introduced, and the indications are better defined to apply them in urgent situations as well as different surgical scenarios.

**Fig. 1:**
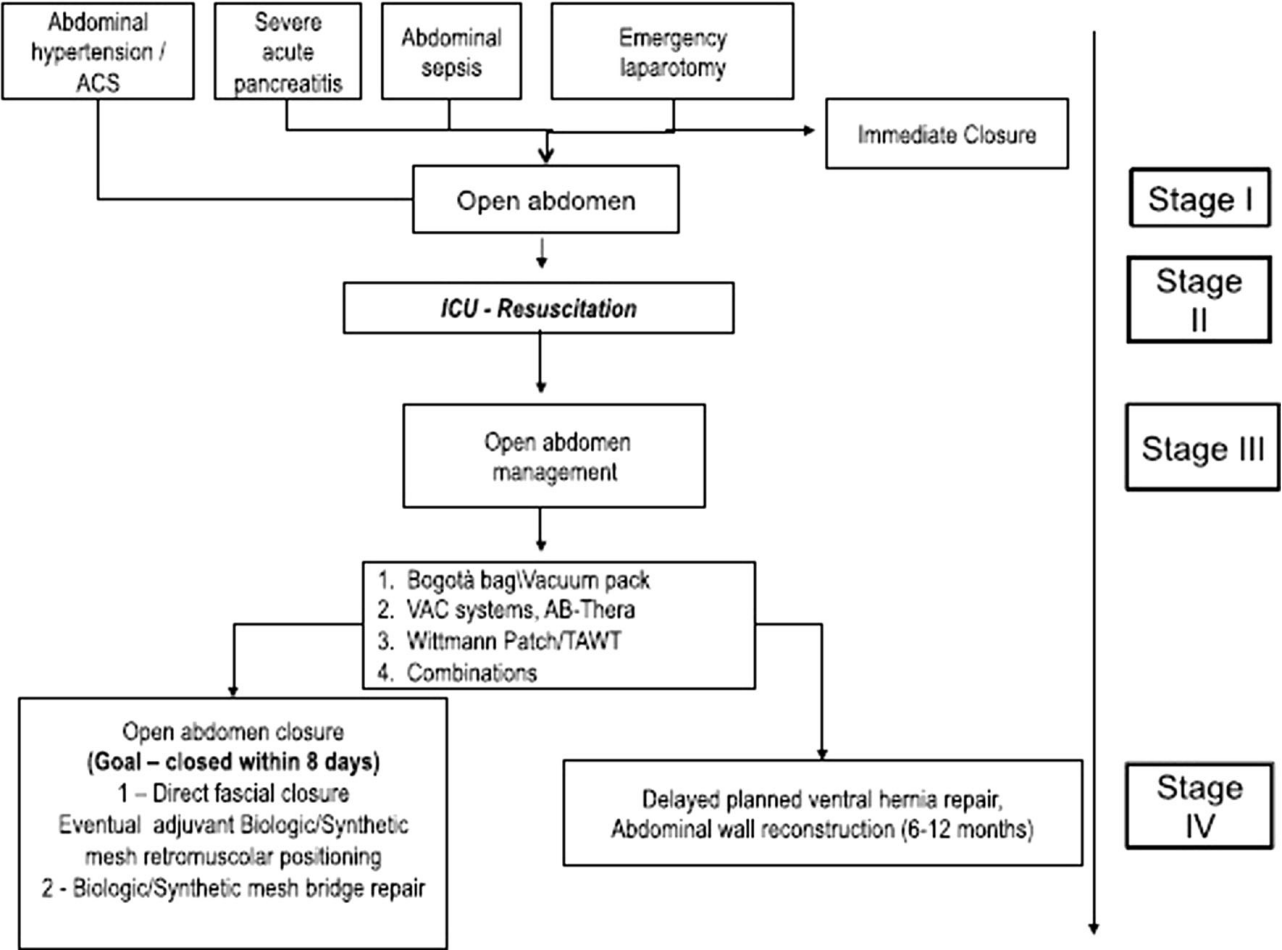
DCS flow chart

In 1897, McCosh was the first surgeon to describe an OA technique for the treatment of generalised peritonitis. He treated these cases, “leaving the abdomen opened, placing surgical sterile drapes amongst bowel and abdominal wall allowing the drainage of peritoneal exudate and peritoneal lavage” [10]. The proposed technique was first abandoned, probably due to the scepticism of scientists, and then revived later when general indications of OA were established by the introduction of the damage control surgery (DCS) concept and its further modifications (Fig. 1).

Novel indications in treatment of OA go beyond severe abdominal trauma, abdominal sepsis, severe acute peritonitis and other conditions that could evolve into abdominal compartmental syndrome (ACS) due to intra-abdominal hypertension (IAH). The World Society of Abdominal Compartment Syndrome (WSACS) defines ACS as “sustained Intra-Abdominal Pressure (IAP) >20 mmHg associated with new onset organ dysfunction” [3]. Mortality associated with ACS is estimated to be between 36 and 80%, respectively, among patients with decompression versus untreated patients.

OA treatment requires the application of TAC.

Ideally, TAC should be easy to perform and rapidly reversible. It should prevent evisceration, preserve abdominal fascia, avoiding retraction of margins, prevent dehydration and adherence formation, drain peritoneal fluids, reduce bacterial load, reduce cytokine rate and permit easy fascia closure [11–20].

In 2007, Acosta et al. described the vacuum-assisted wound closure and mesh-mediated fascial traction technique (VAWCM). They temporarily sutured a polypropylene mesh to fascia medial margins until definitive abdominal closure was achieved according to general clinical/surgical conditions and IAP. In 2011, Acosta et al. published a prospective study in which they demonstrated that in 89% of cases treated with the VAWCM, late fascia closure was obtained with a median of 14 days of OA treatment.

This study aimed to assess survival and risk factors associated with mortality for non-traumatic patients treated with OA and evaluate the outcome in patients treated with OA for ACS and evaluate the fascial closure rate, in-hospital and intensive care unit (ICU) length of stay (LOS) and time to abdominal closure in patients treated with VAWCM.

### Patients and methods

Non-traumatic patients treated with OA at the Policlinico “Paolo Giaccone” at Palermo University Hospital between January 2010 and December 2015 were identified in a prospective database, and the data collected were retrospectively reviewed; patients’ medical and surgical records were collected from charts and the surgical and ICU registries.

The simplified acute physiology score (SAPS II) was collected from the ICU registry and applied according to score indication.

IAP was measured with the intra-vesical technique using the Unometer™ Abdo-pressure™ (ConvaTec Inc., Greensboro, USA) at least every 6 h.

Diagnosed ACS was treated with surgical decompression. For some patients, OA treatment was performed to prevent ACS. For the treatment of patients with OA, Negative Pressure Suprasorb^®^ CNP P1 (Lohmann & Rauscher, Rengsdorf, Germany), with a continuous negative pressure between 25 and 125 mmHg, was applied. Dressing changes were performed every 48–72 h in the operating room. Patients were treated with VAWCM or negative pressure wound therapy (NPWT) with only vacuum-assisted wound closure (VAWC).

The fascia was closed at the end of the OA treatment with running sutures using glycolide and trimethylene carbonate. When fascia closure was impossible due to the fascia retraction or to loss of domain, a planned ventral hernia was performed. Classification of the OA was done according to Björck’s classification.

To perform the VAWCM, the “modified” Acosta temporary abdominal closure (TAC) technique was adopted. The originally proposed technique involves a continuous running suture of polypropylene mesh along the entire length of the medial margins of the rectal fascia. Then, it is longitudinally cut and re-sutured, with a gradual overlap of the margins until the IAP, the oedema of the viscera and the general surgical conditions allow the fascial suture in the midline and the entire removal of the previously positioned prosthesis.

Our approach considers the opening of the anterior rectus sheath and the positioning of the polypropylene mesh inside the muscle cavity at approximately two centimetres under the muscle plane, fixed at the posterior layer. The mesh is positioned during the first dressing change, 48–72 h after the initial surgical operation. The medial margin is then sutured to reconstruct the integrity of the anterior rectus sheath. The mesh is thus longitudinally cut and re-sutured following the principles of the Acosta technique. At the end of the treatment, instead of the complete removal of the prosthesis, it is cut near the medial margin of the sheath, leaving a two-centimetre-wide strip anchored to the sheath. The strip increases the strength of the fascia in the midline, as support for the continuous running suture for abdominal wall reconstruction and closure.

The modified Acosta technique follows the principle of abdominal wall reconstruction with the use of polypropylene meshes positioned underneath even though they are medially fixed instead of laterally fixed in this case.

No short- and long-term complications were recorded due to the modified technique adopted.

## Statistics

Data were analysed in Excel 2013 and IBM SPSS software, version 21. The median was obtained for continuous variables. Comparison of continuous variables was made using Student’s *t* test or Mann–Whitney test, where appropriate. Comparison of categorical variables was made with the Chi-squared (*χ* ^2^ *)* test or Fisher’s exact test. The statistical significance level was set to *p* value <0.05. Univariate analysis for survival was performed; the clinical variables included were age, SAPS II score, sex, ACS, cardiovascular disease (CVD) and mesh-mediated fascial closure. The variables with *p* values <0.05 in univariate analysis were included in the multivariate logistic regression, considering odds ratios with 95% confidence intervals and *p* values <0.05.

## Results

Between January 2010 and December 2015, 96 patients were identified as receiving OA treatment. Sixty-six (69%) were male, with a median age of 69 (40–78) years, and 48 (50%) patients had ACS (Table 1). Sixty-nine patients (72%) underwent VAWCM (Table 2). Bowel obstruction was the most common diagnosis (33%), followed by necrotic haemorrhagic acute pancreatitis (19%) (Table 3). Forty-one (42%) patients were classified as grade 1A OA, 23 (24%) as 1B, 14 (14%) as 1C, 10 (10%) as 2A, 5 (5%) as 2B, 3 (3%) as 2C, 1 (1%) as 3B and 1 (1%) as 4.

**Table 1:** Patients characteristics

Age	Median (range)	69 (40–78)
Sex	M/F	66/30 (69/31%)
Comorbidity	Cv	34 (35%)
	Hypertension	66 (69%)
	Pulmonary	18 (19%)
	Malignant	12 (12%)
	Diabetes	6 (6%)
	Neurological disease	4 (4%)
	Liver failure	4 (4%)
	Renal failure	4 (4%)
	Vascular disease	12 (12%)
Indications for OAT	ACS	48 (50%)
	Prophylactic	26 (27%)
	2nd look	8 (8%)
	Full-thickness dehiscence	14 (15%)

**Table 2:** Type of TAC

All cases	96
VAWC	27 (28%)
VAWCM	69 (72%)

**Table 3:** Surgical diagnosis

All cases	96
Pancreatitis	18
Bowel obstruction	32
Bowel perforation	24
Sepsis	8
Mesenteric ischaemia	14

Forty-eight patients had OA performed for ACS with IAP measured with median peak values of 30 (20–45) mmHg. Types of organ failure present in the ACS patients were renal (*n* = 15), respiratory (*n* = 12), cardiovascular (*n* = 8), combined respiratory and renal failure (*n* = 8) and combined respiratory and cardiovascular failure (*n* = 5). The OA technique was performed at a median of 72 (24–120) h after the primary surgical operation due to the need for a second look (*n* = 8) and full-thickness wound dehiscence (*n* = 14). Twenty-six patients were left prophylactically open at the end of the primary surgical operation. Seventy-nine patients were treated with VAWCM, and 27 patients were treated with VAWC.

Forty-one (68%) of the patients who survived OA achieved primary fascia closure: 5% treated with VAWC (37 median days) versus 95% treated with VAWCM (10 median days) (*p* = 0.0003). Nineteen (32%) were left with a planned ventral incisional hernia (Table 4).

**Table 4:** Outcome of patients with VAWC versus VAWCM treatment

	All	VAWC	VAWCM	*p* value
*N*	96	27 (28%)	69 (72%)	
Survival	60 (62%)	10 (37%)	50 (72%)	0.0012
Dead	36 (38%)	17 (63%)	19 (28%)	
Age	69 (40–78)	70 (40–78)	55 (40–78)	0.2700
Male	66 (69%)	15 (55%)	51 (74%)	0.0810
Female	30 (31%)	12 (45%)	18 (26%)	
Saps II	40 (14–82)	50 (19–82)	35 (14–77)	0.0002
Los in *H*	30 (1–105)	40 (1–105)	25 (1–105)	0.0063
Los in ICU	18 (1–70)	32 (1–70)	11 (1–70)	0.0520
Days on ventilator	15 (1–60)	21 (1–50)	7 (1–60)	0.0077
Fascial closure rate	41 (68%)	2 (5%)	39 (95%)	0.0003
Incisional hernia	19 (32%)	8 (42%)	11 (58%)	

The median number of dressing changes before ending treatment with OA or death was 10 (1–35). Patients with primary fascial closure could be closed after 30 (9–105) days, with a median of 82 (60–105) days for VAWC patients and 12 (9–35) days for VAWCM patients (*p* = 0.0025).

LOS in hospital was 30 (1–105) days. LOS in ICU was 18 (1–70) days, which was required for all 96 patients with a median SAPS II score of 40 (14–82). The surviving group had a median SAPS II score of 37 (14–82) compared to 60 (20–82) among the non-survivors group (*p* = 0.0038). All patients required mechanical ventilation support with a median of 15 (1–60) days, of which 48 were in the ACS group with a median of 21 (1–50) days compared to 11 (1–60) days for the “other reasons for OAT” group (*p* = 0.006).

Sixty (62%) patients survived to OA treatment with a primary fascial closure rate of 68%. Non-survivors (38%) had a median of 2 (1–4) organ failures. Deaths were related to the organ failure, but no deaths were attributed to the OA technique. Forty-eight patients underwent OA treatment due to ACS, and 24 patients (50%) survived compared to the 35 (75%) of those that underwent OA for other reasons (*p* = 0.01). Nevertheless, the ACS group required longer mechanical ventilator support, LOS in hospital and in ICU and had higher SAPS II scores (Table 5).

**Table 5:** Outcome of patients due to the reasons of OA treatment

	All	ACS	Other reasons	*p* value
*N*	96	48	48	
Survival	60 (62%)	24 (50%)	36 (75%)	0.0100
Dead	36 (38%)	24 (50%)	12 (25%)	
Age	60 (40–78)	71 (40–78)	65 (50–78)	0.2600
Male	66 (69%)	32 (67%)	34 (71%)	0.6600
Female	30 (31%)	16 (33%)	14 (29%)	
Saps II	40 (14–82)	49 (19–82)	32 (14–77)	0.0002
Los in *H*	30 (1–105)	32 (1–105)	27 (1–105)	0.0050
Los in ICU	18 (1–70)	20 (1–70)	12 (1–70)	0.0470
Days on ventilator	15 (1–60)	21 (1–50)	11 (1–60)	0.0060

In a univariate analysis of predictors for mortality, ACS, SAPS II, sex, pre-existing CVD and surgical technique (VAWC vs VAWCM) increased the risk of death (Table 6). In the multivariate logistic regression analysis, the same variables were demonstrated to be statistically significant predictors for in-hospital mortality as follows: SAPS II (*p* value 0.004; OR 0.97; 95% CI 0.95–0.99); sex (*p* value 0.01; OR 0.27; 95% CI 0.09–0.8); ACS (*p* value 0.013; OR 2.18; 95% CI 0.79–6.04); pre-existing CVD (*p* value 0.0007; OR 6.60; 95% CI 2.22–19.63); surgical technique (VAWC vs VAWCM) (*p* value 0.0009; OR 0.15; 95% CI 0.05–0.46).

**Table 6:** Prediction of mortality

	All	Survived	Dead	Univariate			Multivariate		
				OR	95% CI	*p* value	OR	95% CI	*p* value
*N*	96	60 (62%)	36 (38%)						
Age	69 (40–78)	65 (40–78)	72 (47–78)	0.9919	0.9593–1.0257	0.636	1.0092	0.9729–1.0469	0.623
Saps II	40 (14–82)	37 (14–82)	60 (20–82)	0.9698	0.9498–0.9902	0.003	0.9681	0.9469–0.9897	0.004
Male	66 (69%)	46 (77%)	20 (55%)	0.3804	0.1564–0.9253	0.033	0.2713	0.0919–0.8013	0.018
Female	30 (31%)	14 (23%)	16 (45%)						
ACS	48 (50%)	24 (40%)	24 (67%)	3.0000	1.2641–7.1198	0.012	2.1823	0.7887–6.0383	0.013
Other reasons	48 (50%)	36 (60%)	12 (33%)						
Cv disease	34 (35%)	14 (23%)	20 (55%)	4.1071	1.6886–9.9900	0.001	6.6032	2.2207–19.6343	0.0007
Other disease	62 (65%)	46 (77%)	16 (45%)						
VAWCM	69 (72%)	50 (83%)	19 (53%)	0.2235	0.0871–0.5739	0.001	0.1498	0.0486–0.4615	0.0009
VAWC	27 (28%)	10 (17%)	17 (47%)						

The Acosta modified technique we suggested showed the absence of short- and long-term complications such as SSI, seroma formation, evisceration, intra-abdominal abscesses, haemorrhages and sepsis, allowing the abdominal wall closure.

Incisional hernia was then recognised through physical examination and radiological findings (computed tomography [CT] scan).

## Discussion and conclusions

The damage control surgery (DCS) concept was introduced in 1983 by Stone and then re-interpreted in 1993 by Rotondo and Scwhab and was first used to improve the prognosis of severe abdominal trauma [21, 22].

It is made up of three treatment stages: first, an urgent surgical operation is performed to find and control bleeding and/or infective sources through abdominal packing techniques, systematic peritoneal cavity exploration and lavages. Then, OA is performed to avoid ACS and to allow further re-operations, protecting abdominal fascia integrity. ICU transfer represents the fundamental phase of the treatment to obtain stabilisation and improvement of vital parameters. The last stage is the gold standard surgical procedure and the definitive fascia closure.

TAC represents an easy method of facilitating re-operations.

In 2009, Koperna et al. evaluated the importance of performing a re-laparotomy in 48 h versus after 48 h, finding that the mortality rate was 28 versus 76.5%. This evidence demonstrates the relevance of the DCS and of the OA treatment instead of re-laparotomy on demand [15]. OA treatment with the postponement of definitive surgery allows a rapid surgical operation with the purpose of DCS. It allows systematic reviews of the abdominal cavity with repeated peritoneal lavages and drainage of abdominal secretions. This procedure can reduce bacterial load and cytokine rate and allows planned abdominal fascia closure, thereby avoiding ACS development. Bleszynski et al. evaluated the mortality risk between patients with a predicted mortality rate of 45% through APACHE-IV score treated with re-laparotomy on demand or OA. Data showed a mortality risk of 38.6 versus 22.8% [16–18].

**Fig. 2:**
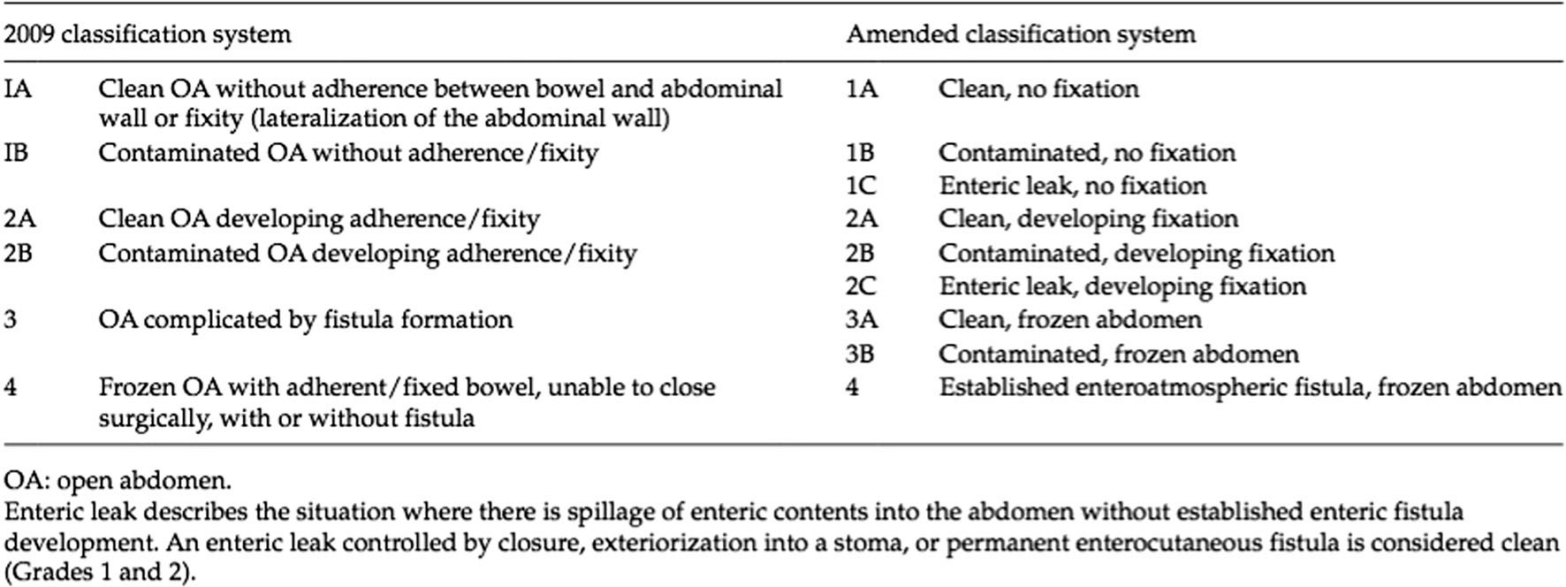
Björck classification

In 2009, Björck et al. [23] proposed a classification of OA to standardise the treatment of abdominal sepsis. This classification was then reviewed in 2016 (Fig. 2). OA treatment requires the application of a TAC. Initial examples of TAC were the skin-only closure and the Bogotà bag. Both of these techniques are characterised by the ease and rapidity of their performance. They are also low-cost procedures. The most important disadvantage is the retraction of fascial margins.

A solution for fascia margin retraction was found between the late 1980s and the early 1990s with the appearance of meshes and the Wittmann Patch technique.

Finally, the vacuum pack seemed to solve the problem of abdominal secretions and their drainage. It consists of the placement of a polyethylene dressing in contact with the viscera, which is covered by surgical drapes and iodine dressings and connected to the vacuum system with a negative pressure of 100–150 mmHg. The evolution of this technique is the NPWT associated with the OA technique [24–26].

In 2012, Roberts et al. conducted a meta-analysis that compared 2 randomised controlled trials and 9 cohort studies (3 prospective and 6 retrospective). They demonstrated that the OA treatment with NPWT compared with other possible TAC has a lower mortality rate (18 vs 27%), major late closing fascia rate (60 vs 52%), lower lactate serum levels and IAP and a shorter LOS.

In recent years, several alternative techniques have been published to optimise the OA technique and to prevent the retraction of fascia margins. In 2007, Acosta et al. described the VAWCM, which consists of the temporary suturing of a polypropylene mesh at fascia medial margins until abdominal definitive closure is permitted according to general clinical/surgical conditions and IAP. Subsequent dressing changes allow systematic review of the abdominal cavity according to NPWT and OA as well as the possibility to gradually accost the medial margins of the abdominal fascia. In 2011, Acosta et al. published a prospective study demonstrating that in 89% of cases treated with the VAWCM, late fascia closure was obtained with a median of 14 days of OA treatment. Furthermore, fewer re-explorations and a shorter duration of open abdominal management are associated with higher fascial closure rates [27].

In 2005, Miller et al. [28] demonstrated that prolonging the OA until the 8th to 9th postoperative day increases the probability of developing complications to nearly 25%. The primary complications described are ACS, SSI and entero-atmospheric fistula (EAF). The latter is considered the worst complication that occurred frequently in severe sepsis syndrome [29–34].

The present study confirms that OA is the gold standard technique for the application of DCS principles. The observed survival rate was 62%, which is similar to other results observed in scientific literature: Acosta et al. [35] reported a survival rate of 70%, Carlson et al. [36] reported a rate of 72%, and Seternes et al. [11] reported a rate of 68%.

ACS, SAPS II, sex, pre-existing CVD and surgical technique (VAWC vs VAWCM) increased the risk of death. In the multivariate logistic regression analysis, the same variables were demonstrated to be statistically significant predictors for in-hospital mortality.

The reason for the use of the OA technique was ACS in 50% of cases, with a survival rate of 50%, confirming that ACS is a life-threatening condition.

Primary fascial closure was obtained in 68% of cases, and only 32% required a planned incisional hernia. Roberts et al. adopted NPWT as the gold standard procedure.

The surgical technique described above has been demonstrated to have high potential for widespread adoption when VAWCM is required. It summarises the Acosta principles regarding the performance of mesh-mediated fascial traction in order to avoid the retraction of fascial margins. It also follows the principles of abdominal wall reconstruction by the positioning of a polypropylene mesh underlay. The mesh increases the strength of the fascia in the midline, providing support for the continuous running suture for abdominal wall reconstruction and closure. No short- and long-term complications were described in the series proposed with the highest closure rate, 95 versus 5% of patients with VAWC, and with lower median days for primary fascial closure, a higher survival rate and a shorter LOS in hospital and ICU.

In addition to the results obtained by the applied indication of the OA technique and the chosen TAC strategy, several other aspects influence patient outcomes. Management of severe sepsis and septic shock requires a multidisciplinary team to assess resuscitation, respiratory support and infection control.
